# Bidirectional Influences of Information Sampling and Concept Learning

**DOI:** 10.1037/rev0000287

**Published:** 2021-07-19

**Authors:** Kurt Braunlich, Bradley C. Love

**Affiliations:** 1 Department of Experimental Psychology, University College London; 2 Laboratory of Brain and Cognition, National Institute of Mental Health, Bethesda, Maryland, United States; 3 The Alan Turing Institute, London, United Kingdom

**Keywords:** decision making, categorization, attention, active sampling

## Abstract

Contemporary models of categorization typically tend to sidestep the problem of how information is initially encoded during decision making. Instead, a focus of this work has been to investigate how, through selective attention, stimulus representations are “contorted” such that behaviorally relevant dimensions are accentuated (or “stretched”), and the representations of irrelevant dimensions are ignored (or “compressed”). In high-dimensional real-world environments, it is computationally infeasible to sample all available information, and human decision makers selectively sample information from sources expected to provide relevant information. To address these and other shortcomings, we develop an active sampling model, Sampling Emergent Attention (SEA), which sequentially and strategically samples information sources until the expected cost of information exceeds the expected benefit. The model specifies the interplay of two components, one involved in determining the expected utility of different information sources and the other in representing knowledge and beliefs about the environment. These two components interact such that knowledge of the world guides information sampling, and what is sampled updates knowledge. Like human decision makers, the model displays strategic sampling behavior, such as terminating information search when sufficient information has been sampled and adaptively adjusting the search path in response to previously sampled information. The model also shows human-like failure modes. For example, when information exploitation is prioritized over exploration, the bidirectional influences between information sampling and learning can lead to the development of beliefs that systematically differ from reality.

At its heart, category learning involves extracting key patterns that capture the essence of our experiences, and allow us to make accurate inferences about the external world. Two fundamental challenges for psychological research are to understand how this knowledge is acquired, and once acquired, how it can be flexibly used to guide successful interactions with the external world. Although successful categorization models differ in how categories are represented in memory (e.g., as flexible clusters, exemplars, or prototypes; Love et al., 2004; Nosofsky, 1986; Smith & Minda, 1998), they tend to sidestep the question of how sensory information is encoded, but assume that the representations considered during deliberation are available to the decision maker, and can be modulated by selective attention based on their behavioral relevance (Figure 1A). Attention plays a key role in allowing these models to capture the flexibility by which humans are able to organize stimuli into categories (Shepard et al., 1961). These attentional weights provide key information about how different sources of information are organized during decision making.[Fig fig1]

Leading contemporary categorization models, therefore, tend to treat category decisions as “single-step” decision problems; in which agents make decisions about the final choice, but not about what information to sample. Although it is plausible that decision makers encode all relevant stimulus information from the low-dimensional stimuli typically considered in the laboratory,^1^ in high-dimensional environments, encoding all available sensory information is inefficient, and can impair learning. This reflects a fundamental computational constraint (known as the *curse of dimensionality*), which affects both machine-learning algorithms (Hastie et al., 2009; Li et al., 2017) and human decision makers (e.g., Bulgarella & Archer, 1962; Edgell et al., 1996; Pishkin et al., 1974; Vong et al., 2019).

To be able to interact efficiently in high-dimensional environments, humans actively sample information from sources expected to provide behaviorally relevant information (e.g., Cook et al., 2011; Markant et al., 2015; Markant & Gureckis, 2014; Nelson & Cottrell, 2007; Yang et al., 2016). This is apparent, not only for decisions involving the collection of discrete samples of information across extended periods of time^2^ but also are reflected in subtle patterns of eye movements during decisions of relatively short duration (i.e., less than 3 s; Blair et al., 2009; Rehder & Hoffman, 2005a). This partially reflects fundamental constraints of extrafoveal visual acuity, which drive decision makers to integrate sensory information through a series of saccades to different locations. As expectations about ones environment can change based on the values of incoming sensory information, approaches that treat categorization decisions as single-step problems tend to ignore attentional effects that can occur within individual trials (Blair et al., 2009; Gottlieb, 2018; Gottlieb & Oudeyer, 2018).

Through experience, decision makers learn to selectively attend to behaviorally relevant stimulus features (Shepard et al., 1961). When observations are drawn randomly (as in typical laboratory experiments), participants tend to gain equivalent experience with all possible stimulus configurations. When participants are free to select the stimuli from which they learn, however, they tend to selectively sample information to test specific hypotheses (this is known as *hypothesis-dependent learning bias*; Markant & Gureckis, 2014). This can facilitate learning when the generated hypotheses closely resemble the true structure of the environment, but can otherwise impair learning. Humans often show a bias for testing simple hypotheses, for example, and this can impede learning (and/or lead to the development of inaccurate beliefs) when the true structure of the world is complicated.

Here, we develop a computational model, Sampling Emergent Attention (SEA), designed to reflect this effect. The model actively selects information as a function of its goals and its current beliefs, and learns to selectively sample information sources expected to provide behaviorally relevant information.^3^ Leading contemporary models of categorization (e.g., Kruschke, 1992; Love et al., 2004; Nosofsky, 1986), in contrast, tend to sidestep the question of how information is initially sampled. Rather than investigating how decision makers choose what information sources should be sampled, these models emphasize questions related to how stimulus information is organized. A particular focus of these models is to understand how, through selective attention, stimulus representations are contorted such that representations of behaviorally relevant stimulus dimensions are accentuated (or “stretched”), and representations of irrelevant dimensions are ignored (or “compressed”). We therefore describe these models as treating categorization decisions as “single-step” decision problems (Figure 1A), as decisions are made about the final choice, but sequential intratrial active sampling behavior (Figure 1B) is not considered.

SEA consists of two interacting components; each of which can be considered normatively optimal in its own right. The first component reflects the decision maker’s beliefs and expectations about the environment, and the second estimates the value of different information states. Interactions between the two components allow the model to select samples that maximize expected utility. Whereas contemporary categorization models often include attentional parameters that “contort” stimulus representations along perceptually separable stimulus dimensions (Figure 1A; Garner, 1976),^4^ the proposed model reconceptualizes attention as the expected gain in utility from sampling particular information sources. Despite this fundamental difference, SEA predicts classic behavioral effects associated with selective attention (Nosofsky et al., 1994; Shepard et al., 1961). Its active sampling behavior additionally closely resembles patterns of human eye movements (e.g., Blair et al., 2009; Rehder & Hoffman, 2005a).^5^ Finally, like human learners, SEA can also develop inaccurate beliefs about the world when it fails to balance demands for exploration and exploitation (Rich & Gureckis, 2018).

## Optimal Experiment Design and Human Information Sampling

Several groups have used the principles of optimal experimental design (OED; Fedorov, 1972, 2010; MacKay, 1992) to investigate whether humans strategically sample information to test specific hypotheses. Although the calculations underlying OED can be computationally prohibitive for cognitively limited human decision makers, these studies indicate that, despite being susceptible to perceptual (Itti et al., 1998; Yamada & Cottrell, 1995; Zhang et al., 2008) and decisional (Klayman, 1995) biases, we are often able to select information samples that resolve uncertainty about specific hypotheses. This effect is apparent both during the performance of traditional categorization tasks (Markant et al., 2015; Markant & Gureckis, 2014), and during naturalistic behavior. Preschool children, for instance, spontaneously conduct “experiments” to test specific causal hypotheses about the objects they are playing with (Cook et al., 2011). Hypothesis-dependent sampling strategies have also been identified through study of human eye movements. During categorization, for instance, we tend to selectively fixate on stimulus locations that resolve uncertainty about the potential category label (Nelson & Cottrell, 2007; Yang et al., 2016). During visual search, we similarly tend to fixate on locations expected to maximize information about the target location (Najemnik & Geisler, 2005).

To select useful information sources to sample, a decision maker must be able to simulate future events. This capacity for *preposterior* analysis^6^ involves predicting the probability and utility of future states. When diagnosing a patient, for instance, doctors must have sufficient knowledge of human pathology to identify plausible diagnoses. They must also be able to use this knowledge to select medical tests that efficiently differentiate between the most probable diagnoses. To reflect the fact that some some results can be more informative than others,^7^ full preposterior analysis aggregates information about both the probability and usefulness of each expected result. In practice, this forward-search process can be computationally prohibitive for large problems, necessitating an approximation to the full preposterior search performed by SEA.

## What Is a “Useful” Question?

A number of different sampling norms have been used to define the usefulness of sampling a particular dimension (see Nelson, 2005). *Disinterested* sampling norms seek to maximize decision accuracy. One way to define the usefulness of a particular medical test, for instance, would be the degree to which it is expected to improve the probability of making a correct diagnosis.^8^ In contrast, *situation-specific* sampling norms maximize reward rather than accuracy, and may be preferable when payoffs are asymmetric (i.e., when the maximization of accuracy differs from the maximization of reward; Meder & Nelson, 2012). For example, incorrectly diagnosing a malignant tumor as benign can be more costly than incorrectly diagnosing a benign tumor as malignant.

Utility-sensitive decision makers should also consider the costs associated with sampling each information source. Invasive medical tests (e.g., a biopsy), for example, can be more informative than noninvasive tests (e.g., an X-ray). As a result, doctors must determine whether the benefits of a particular test are outweighed by its cost. A purely exploitative decision maker should stop deliberating and commit to a choice when the expected gain in value from a particular test is outweighed by its cost. An exploratory decision maker, however, might be willing to tolerate a small cost to learn about the environment. Agents must, therefore, carefully balance demands for exploration and exploitation when learning about a domain, or risk developing inaccurate beliefs (as depicted in Figure 2). Although medical decisions are often extended in time, we face the same challenges when making rapid decisions (i.e., deciding what information should be sampled), even about which eye movements to make, as evaluated in category learning experiments.[Fig fig2]

## Self-Termination and Branching

As its beliefs are updated after observing each sample, SEA can display “branching” and “self-termination.” Branching involves changes in sampling strategy based on the values of the incoming information. Self-termination occurs when decision makers decide to commit to a choice, rather than selecting additional samples.

**Figure 1 fig1:**
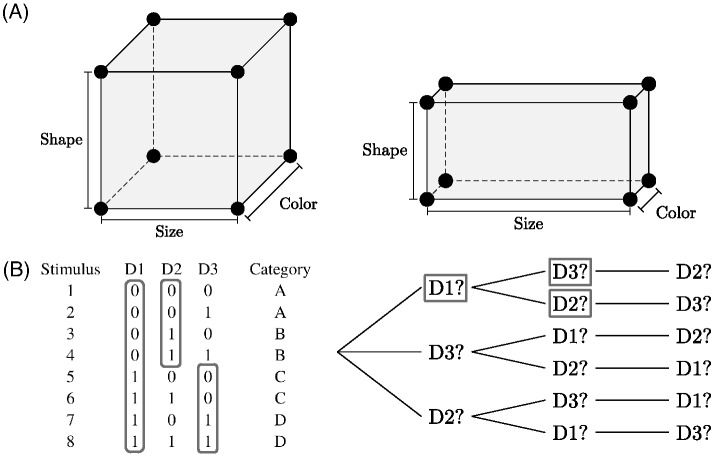
Two Views of Attention *Note*. (A) Contemporary categorization models tend to sidestep questions related to how decision makers sample information from the world. Instead, their emphasis is on how multidimensional stimulus representations are are “contorted” by selective attention (e.g., Kruschke, 1992; Love et al., 2004; Nosofsky, 1986). In the example on the left, three stimulus dimensions (Size, Color, and Shape) are equally attended. On the right, “Size” is given greater attentional weight than “Shape” or “Color”. (B) Active sampling requires decisions, not only about the appropriate final choice but also about what samples should be selected. In the category structure depicted at left (Blair et al., 2009), the optimal sampling strategy is to first sample Dimension 1, and then, depending on its value, sample either D2 or D3 (gray rectangles denote informative samples). This temporally ordered sequence is illustrated at right. It is never necessary to sample all three dimension if D1 is sampled first.

Such decisions about *when* to commit to a choice are a fundamental component of many temporally extended decisions (Figure 1B). Decision makers may fail to capitalize on transient opportunities for reward (or accrue excessive costs associated with deliberation) if they wait too long before committing to a choice. Conversely, if they respond too quickly, they may fail to collect enough evidence to support a desirable level of accuracy. We propose that the depth of forward search, which varies from myopic search to full preposterior analysis (Figure 1B), can be adjusted based on contextual demands on response timing. As clusters are “activated” based on the observed features, and the cluster representations predict the appropriate final choice (e.g., the category label), as more information is accumulated/sampled, inferences about the correct response become more accurate (assuming that clusters reflect relevant aspects of the environment).

Several models have been proposed to address the question of self-termination. “Integrate-to-bound” models, such as the Sequential Probability Ratio Test (SPRT; Wald & Wolfowitz, 1948) and the Drift Diffusion Model (DDM; Ratcliff, 1978), for example, operate by collecting evidence for competing hypotheses over time (in the form of a log-likelihood ratio), and committing to a choice when the strength of the cumulative evidence exceeds a predefined threshold. In typical implementations of these models, the threshold remains stationary during deliberation, and is chosen to balance the trade-off between accuracy and deliberation cost. Unlike SEA, however, these models act as passive observers, as they do not select the samples from which they learn.

In contrast, SEA selects samples sources of information through consideration of its beliefs about the environment, and updates these beliefs following the observation of each sample. Incidentally, the calculations involved in this procedure provide a principled way to define the termination criterion. Although a purely exploitative decision maker should commit to a choice when the expected gain in utility for each sample is outweighed by its cost, an exploratory decision maker may be willing to bear some sampling cost to learn about the environment. Whereas the DDM and SPRT define the termination criterion to balance demands for accuracy with missed opporunity costs, in SEA the termination criterion is calculated with regards to expected information gain, and a heuristic that strives to balance the trade-off between exploration and exploitation.^9^

Because SEA strives to sample the most informative information source at each step, successive samples tend to become less informative. Concurrently, costs associated with deliberation tend to accrue. The likelihood of committing to a final choice, therefore, tends to increase with the number of samples observed. The dynamic nature of this decision boundary resembles that of some integrate-to-bound models (e.g., Cisek et al., 2009; Niyogi & Wong-Lin, 2013; Standage et al., 2013; Thura et al., 2012), which have been developed to account for modulation of the speed-accuracy trade-off during decision making. In both frameworks, the collection of additional information (which can be perceptual and/or mnemonic) generally improves decision accuracy, but also tends to increase costs associated with deliberation. However, whereas integrate-to-bound models tend to describe the decision process as the diffusion of a variable through time, SEA tracks expected information gain in conjunction with the accruing costs associated with collecting information samples. SEA additionally proposes that the depth of decision planning (i.e, ranging from myopic to full-preposterior forward search) influences the trade-off between decision accuracy and cost.

As discussed above, although leading contemporary models provide a compelling account for how decision makers organize information during decision making, they tend to sidestep questions relating to how decision makers choose information sources to sample, how they sequentially update their representations during deliberation, and how they terminate this deliberative process (for experimental evidence of sequential processing during human categorization, see: Milton & Wills, 2004; Wills et al., 2015). There are, however, a few notable exceptions. The Exemplar-Based Random Walk model (EBRW; Nosofsky & Palmeri, 1997), for example, sequentially retrieves items from memory until the available evidence exceeds a decision threshold. The EBRW does not, however, selectively encode stimulus information, but rather initially encodes all stimulus representations considered during the decision. Similarly, the extended generalized context model (EGCM-RT; Lamberts, 2000) stores representations of individual exemplars, but sequentially encodes stimulus feature values. As the feature values are encoded, the similarity between the stimulus and exemplars stored in memory is updated. This process resembles the sequential sampling problem faced by human learners, but information sampling is not strategic (i.e., it does not reflect previously retrieved information). In addition, the EGCM-RT will consider all stimulus features instead of self-terminating.

## The Proposed Model

Here, we introduce a novel model of categorization, SEA, which is designed to treat decision making as an active sampling problem (in which decisions are made, not only about the final choice (Figure 1A), but also about what information to sample; Figure 1B). It combines two normatively motivated components. The first is a concept-learning component that reflects the decision maker’s knowledge of the world. The second is a utility-sensitive sampling component that calculates the expected utility of particular states. The two components interact to perform preposterior analysis. These interactions allow the model to selectively sample from information sources that are expected to be useful for differentiating a set of “active” hypotheses.

**Figure 2 fig2:**
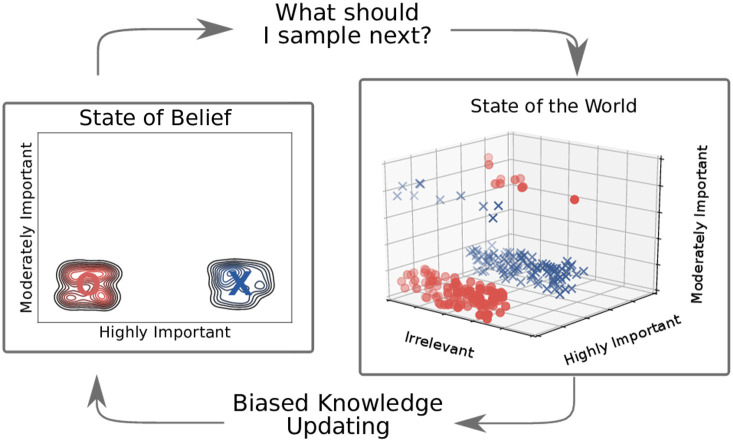
Bidirectional Influences of Information Sampling and Learning *Note*. In this example, a decision maker has learned to categorize stimuli, which vary according to three dimensions (one that is highly informative, one that is moderately informative, and one that is irrelevant), into two categories (denoted by blue crosses and red circles) by actively sampling information from the external world (110 stimuli, *randomly* drawn from this imaginary world, are illustrated at the right). Their knowledge of the world (depicted as two probability distributions at left) reflects the samples that have been observed. In this example, the decision maker has learned that the “highly important” dimension predicts the category label, but has not learned that the “moderately important” dimension mediates this relationship. As a result, this learner would be unable to classify all stimuli accurately. Characteristics of the external world (e.g., costs associated with sampling each dimension, or costs associated with incorrect choices), as well as characteristics of the learner (e.g., some learners might show a stronger bias for simple hypotheses) influence what is ultimately learned. See the online article for the color version of this figure.

Strategically sampling learners, such as SEA, can easily learn representations that deviate from reality (Figure 2; Rich & Gureckis, 2018). This can happen when the learner fails to balance demands for exploration and exploitation. For instance, when a number of costly experiences with a stochastic variable are encountered early in training, a cost-sensitive decision maker may choose to avoid it, and never learn that it actually yields net long-term gain.^10^ To encourage exploration of undersampled information sources, SEA can include exploration bonuses for undersampled information sources. As the partially observable Markov decision process (POMDP) can only be solved for relatively simple problems (Knox et al., 2012), this mechanism can be seen as a heuristic linking the concept-learning and utility-sensitive sampling components.

Although category learning with feedback is typically treated as a supervised learning task, the present work recasts it as a problem in which the agent learns to traverse a series of probabilistic states (i.e., information samples) while minimizing sampling costs and maximizing reward (similar to reinforcement learning; Kaelbling et al., 1996; Sutton, 1990). Although SEA will initially sample uniformly across dimensions, it will gradually learn to sample selectively from dimensions expected to provide useful information. The resulting representational structure is efficient, in that it minimizes both the amount of information encoded across experiences, and the amount of information considered during individual decisions.

In SEA, the effects of selective sampling emerge with learning, and so sampling strategies change as the model learns about the environment. These bidirectional interactions between information sampling and concept-learning result in high-density representations along dimensions SEA believes are useful, and low-density representations along dimensions SEA deem irrelevant (reflecting the relative sampling frequency of these dimensions). This is analogous to the effects shown in Figure 1A, which are captured by “single-step” categorization models, which sidestep the information sampling stage of decision making, and selectively weight dimensions through attentional processes (e.g., Kruschke, 1992; Love et al., 2004; Nosofsky, 1986). In both frameworks, behaviorally relevant stimulus dimensions have greater influence on the final choice than do irrelevant dimensions.

Active sampling can lead to a self-enforcing pattern of belief updating, where beliefs about the world influence the information that is sampled from it, and this information is used to update beliefs. This can have important consequences on learning efficiency. When decision makers are free to select the stimuli from which to learn, they often learn more efficiently than when stimuli are presented in a predetermined order (Castro et al., 2009; Gureckis & Markant, 2009; Markant & Gureckis, 2010, 2014; Markant et al., 2015). This effect, however, depends on the structure of the problem being learned (Enkvist et al., 2006; Markant & Gureckis, 2010, 2014). Bidirectional interactions between information sampling and learning can also determine *what* concepts are ultimately learned. One example is the blocking effect (Kamin, 1969), wherein after learning that a particular dimension is informative, a decision maker will tend to exploit this knowledge rather than continue to explore other information sources. To avoid these kinds of “knowledge traps” (Rich & Gureckis, 2018), decision makers must successfully balance demands for exploration and exploitation (Kaelbling et al., 1996; Sutton & Barto, 1998).

## Model Overview

**Figure 3 fig3:**
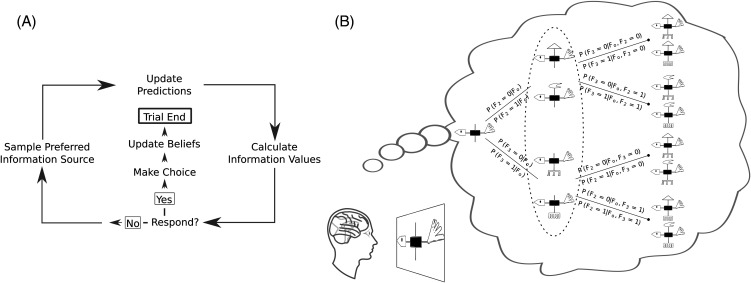
Active Learning and Preposterior Analysis *Note*. Active learning requires decisions, not only about the final choice but also about what information sources should be sampled. (A) When the cost of sampling an information source exceeds the expected gain in utility, a purely exploitative decision maker should commit to a final choice. (B) To decide whether to stop deliberating, or to sample an additional stimulus dimension, SEA performs *preposterior analysis*. In the illustrated example, two of the four features used by Rehder and Hoffman (2005a, that is, the head and tail of an abstract bird stimulus) have been observed, and all possible future sequences of samples are simulated. In typical categorization tasks, participants strive to maximize the accuracy of the final choice (as in Table 1), and the cost of sampling each dimension is equivalent. For other kinds of decisions (e.g., those involving medical diagnoses), outcomes associated with the final choice can be associated with asymmetric values (e.g., the cost of a false negative is often greater than the cost of a false positive). Similarly, different tests can impose different costs (e.g., an MRI is more expensive than a blood test). Our beliefs about costs, values, and the probabilities of future events influence what information is sampled, and therefore what is ultimately learned. *Ellipse*: A decision maker using a myopic planning process would consider the possible results of only a single sample into the future, and then make the best possible response. Full preposterior analysis is generally more accurate, as it also considers the potential results of subsequent samples.

In this section, we present SEA, and its potential variations. SEA’s information-value component determines which (if any) features should be sampled. Its learning component provides the information-value component with the probabilities required to determine the sampling policy, and is updated based on the information sampled. Below, we specify these components, outline their interactions, and consider model variants that incorporate mechanisms that reflect the constraints of human decision makers.[Fig fig3]

### Concept-Learning Component

The concept learning component we use is closely related to the Rational Model of Categorization (RMC; 1991b; Anderson & Matessa, 1990), although any generative probabilistic model would also likely be appropriate. The RMC incrementally learns to sort stimuli into appropriate clusters, and can make near-optimal use of past information during learning and prediction. Here, we provide an overview of the RMC. Additional details can be found in the original articles.

The RMC is a flexible clustering model, which learns to parcelate representational space into clusters based on its experience with the normative characteristics of the task environment. Formally, the probability that any unobserved stimulus dimension, *F*_*i*_, will take a particular value, *j*, can be inferred by weighting the prediction of each cluster, *P*(*F*_*i*_ = *j*|*k*), by the probability of the cluster given the observed features, *P*(*k*|*F*_*O*_):

$$P(F_{i}=j|F_{O})=\sum_{k}P(F_{i}=j|k)P(k|F_{O}).$$ 1

where *P*(*F*_*i*_ = *j*|*k*) is calculated using Equation 2, and *P*(*k*|*F*_*O*_) is estimated using Equation 3. By this notation (which we will use throughout the article) the subscript, *O*, denotes the index of the observed features of a given stimulus, and *i* denotes the index of the considered feature. For instance, given a stimulus (including both observed and unobserved dimensions) defined as vector *F* = [2, 1, 1, 2], if the second feature was under consideration, and the third and fourth features were known, then *i* would be 2, *O* would be [False, False, True, True], and *F*_*O*_ would be [?, ?, 1, 2].

For each dimension, discrete feature values are assumed to be distributed according to a Dirichlet density characterized by dimension-value parameters α_*j*_, and dimension-wide parameters, α_0_ (where α_0_ = Σ_*j*_ α_*j*_). The Dirichlet distribution allows the data to determine the number of clusters (as in SUSTAIN; Love et al., 2004), and allows for a potentially infinite number of clusters. However, between one and three clusters per category is typical. These desirable characteristics of the Dirichlet distribution have led to it being used in many categorization models (e.g., Anderson, 1991a; Griffiths et al., 2007).

Across learning, SEA tracks the number of items in cluster *k* with the same value, *j*, on feature *i* in *C*_*ij*_. The posterior is also Dirichlet-distributed, and the probability that a feature will take a particular value within a cluster is as follows:

$$P(F_{i}=j|k)=\frac{\alpha_{j}+C_{ij}}{\alpha_{o}+\sum_{j}C_{ij}}.$$ 2

As *C*_*ij*_ becomes populated through experience, it exerts stronger influence on *P*(*F*_*i*_ = *j*|*k*) relative to the prior. The prior parameters (the α’s), therefore play an important role during early learning, as they allow SEA to appropriately estimate its uncertainty when few samples have been observed. After a single trial, for example, it would be erroneous to infer that *all* future objects will display the observed values.

Bayes’ theorem can be used to calculate the last term in Equation 1, *P*(*k*|*F*_*O*_). This term represents the probability (or “activation”) of each cluster given the observed features:

$$P(k|F_{O})=\frac{P(F_{O}|k)P(k)}{\sum_{k}P(F_{O}|k)P(k)},$$ 3

where *P*(*F*_*O*_|*k*) is calculated using Equation 2, and *P*(*k*) represents the prior probability that any stimulus will be assigned to cluster *k*. This probability *is* calculated as follows:

$$P(k)=\frac{cn_{k}}{(1-c)+cn},$$ 4

where *c* denotes the coupling probability (a parameter that determines the probability that two objects come from the same category), *n*_*k*_ is the number of items already assigned to cluster *k*, and *n* is the total number of stimuli observed. The prior probability that a stimulus will be assigned to a *novel* cluster is as follows:

$$P(0)=\frac{\left(1-c\right)}{(1-c)+cn}.$$ 5

As no clusters have yet been created on the first trial, the model will start with a single cluster with each feature initialized with a uniform probability of occurring (as in Equation 5). With greater experience, the model will incrementally learn a single partition of stimuli into clusters.^11^ Although the fully normative solution would be to consider all possible partitions of stimuli into clusters (Anderson, 1991a), this approach is intractable for all but the simplest problems.^12^ The incremental approach may also be more psychologically valid (Love et al., 2004). With the parameters set as in the simulations described below, the model tends to sample all features before selectively sampling from those expected to provide useful information.

### Combining Concept-Learning With a Utility-Sensitive Sampling Norm

When facing a choice with an uncertain outcome, the expected utility of a particular action, *a*, can be calculated by weighting the utility of each resulting state by its probability. In a category learning experiment, for example, one category label may be more probable than the other, but yield lesser reward. The action-utility function shown in Table 1 corresponds to a contingency table in which two states (or categories), *s*_*p*_ and *s*_*q*_, are mutually exclusive and exhaustive (i.e., *P*(*s*_*p*_ ∪ *s*_*q*_) = *P*(*s*_*p*_) + *P*(*s*_*q*_) = 1), and the decision maker must choose the appropriate action (*a*_*p*_ or *a*_*q*_; in a categorization experiment, this corresponds to the category label). The table depicts a hypothetical action-utility function reflecting the utility for two actions: *a*_*p*_ and *a*_*q*_. For this particular example, maximizing utility is equivalent to maximizing accuracy, as correct responses are rewarded with 100 utility units, and incorrect responses are awarded zero units. The table could be expanded to include more than two actions and states.[Table tbl1]

For the action-utility function shown in Table 1, the expected utility, E(U) of action *a*_*p*_ can be calculated as follows:

$$E(U(a_{p}))=U(a_{p}|s_{p})P(s_{p})+U(a_{p}|s_{q})P(s_{q}).$$ 6

For example, if *P*(*s*_*p*_) = 0.7, and *P*(*s*_*q*_) = 0.3, the expected utility of choosing *a*_*p*_ would be 70 and that of *a*_*q*_ would be 30. In this case, the utility-maximizing action would be to choose *a*_*p*_. As mentioned, payoffs can also be asymmetric. For instance, if the lower-left entry in Table 1 was −1,000, there would be a high penalty associated with *a*_*p*_ when state *s*_*q*_ holds, and the optimal choice would switch to *a*_*q*_.

The above examples describe problems involving a single feature with two possible values (*s*_*p*_ and *s*_*q*_). Real-world decisions typically require decision makers to integrate evidence across multiple features, which often have more than two possible values. When diagnosing a tumor, for instance, it might be necessary to consider results from blood tests as well as from CT-scans or MRI. Categorization tasks are often designed to reflect this aspect of real-world decisions; participants must integrate information across relevant stimulus features.

In SEA, as in Anderson’s RMC (1991b; Anderson & Matessa, 1990), the category label is treated like any other cluster feature, and Equation 1 can be used to calculate the probability of each label, given the observed feature values. The value of action, *a*, given the observed features, *F*_*O*_,^13^ can be estimated by summing over states, *s*, and subtracting the costs associated with sampling each observed feature, *ℒ_o_*:^14^

$$E(U(a|F_{O}))=\sum_{s}U(a|s)P(s|F_{O})-\sum_{o\in O}{ℒ}_{o},$$ 7

where *P*(*s*|*F*_*O*_) is provided by Equation 1, and *U*(*a*|*s*) was introduced in Equation 6. Before learning about the environment, a uniform prior (resulting from Equations 4 and 5) drives probabilistic sampling of each stimulus feature.

The estimated utility of the current state, *F*_*O*_, can be estimated by maximizing over possible actions:

$$E(U(F_{O}))={\text{argmax}}_{a\in \text{Actions}}(E(U(a|F_{O}))).$$ 8

As discussed, real-world decisions often require decision makers to decide what information should be sampled. This is important, as the information that is sampled can influence the final choice. The results of a blood test, for instance, can influence a doctor’s decision about whether to suggest chemotherapy for a patient. To estimate the utility of a test that reveals the value of an unknown feature (e.g., “cancer antigen present” vs. “cancer antigen absent”), we consider how much the results of the test would improve the utility of the current state (where the current state is defined by the vector of observed features, *F*_*O*_). The expected utility of the state after sampling unobserved feature *i*, can be estimated by summing across its possible values, *j*:

$$E(U(F_{O},F_{i}))=\sum_{j\in F_{i}}E(U(F_{O},j))P(F_{i}=j),$$ 9

where $E(U(F_{O},j))$ denotes the expected utility of the state if value *j* (of unobserved feature *F*_*i*_) was included in the vector of observed features. Equation 9 demonstrates how the expected utility of the state can be calculated for a single feature. As each feature can have multiple values (two in the simulations described below), the model explores each branch for each feature. During “myopic” decisions, the model considers only a single step into the future. Preposterior analysis (which is implemented in SEA as a “depth-first” search process), involves imagining each branch several steps into the future. The computational demands of preposterior analysis, therefore, are high, even for the relatively low-dimensional decision problems commonly considered in the categorization literature.

Equation 9 contributes to the calculation of the gain in utility (cf., Nelson, 2005; Nelson et al., 2010) from sampling unobserved feature *i*:

$$G(F_{i})=E(U(F_{O},F_{i}))-E(U(F_{O})).$$ 10

SEA proposes that this expected increase in utility from sampling *F*_*i*_ is the key variable to consider when deciding what feature to sample, or whether to stop sampling and commit to a final choice. When *G*(*F*_*i*_) for all features is less than, or equal to zero, a cost-sensitive decision maker should stop sampling and commit to a final choice. When G(*F*_*i*_) for at least one feature is greater than zero, an exploitative strategy would be to sample the feature with the greatest expected gain.

Importantly, costs are often dependent across features. The cost of a blood test, for instance, can be substantially less if other blood tests have already been ordered. A normative strategy therefore requires the consideration of all possible sequences of tests to account for these potential dependencies. As the computational demands of this approach increase exponentially with the number of features considered, it can only be justified when decisions involve a low number of stimulus features (as is common in psychology experiments), or when there is sufficient time available for deliberation and the stakes are high.

An alternative would be to select tests myopically, selecting the next test without consideration of those following it. Such selection strategies are guaranteed to be optimal only if the next test happens to be the last. Interestingly, previous work has indicated that human behavior is often myopic during sequential sampling (or deferred decision) tasks (Busemeyer & Rapoport, 1998), which require multiple samples to be drawn from a single noisy stimulus feature (Edwards, 1965; Rapoport & Burkheimer, 1971). Although such tasks similarly require participants to decide, at each time-step, whether to consider additional information or commit to a final choice, the problems considered here require the integration of information across multiple stimulus features. This poses an additional challenge, as decision makers must know which features provide useful information with regards to their goal. In our model, the concept learning component provides this kind of information (i.e., knowledge of the problem’s underlying structure) to the information-utility component (which then identifies the most informative samples).

### Balancing Demands for Exploration and Exploitation

Precisely determining the optimal balance of exploration and exploitation is intractable for most tasks and is only possible for special cases (Kaelbling, 1993; Kaelbling et al., 1996; Simsek & Barto, 2006). To derive the optimal solution, one would need to make several assumptions. It would be necessary, for instance, to estimate the number of trials left in the study (as the negative consequences of choosing a suboptimal strategy increases with the number of trials on which it is applied). It would also be necessary to estimate how rewarding the environment is (as optimal inference requires normalizing estimates based on environmental characteristics). It would also be necessary to estimate the probabilities of different category structures, which represents uncertainty about the appropriate categorization strategy (alternatively, one could restrict the possible forms of the environment, as in Stankiewicz et al., 2006). Finally, it would also be necessary to consider the probability of any of these factors changing over time (cf., Brown & Steyvers, 2009; Gittins & Jones, 1979; Steyvers et al., 2009).

Fortunately, a number of heuristic methods exist (Kaelbling, 1993; Kearns & Singh, 2002; Moore & Atkeson, 1993; Schmidhuber, 1991; Sutton, 1990). We combine two of these heuristic methods: stochastic choice via a softmax choice rule, and exploration bonuses for underexplored options (Kaelbling, 1993). The exploration bonus, *E*, could take many forms. In Kalman filter models, this term often takes the form of an uncertainty bonus that reflects the standard deviation of the choice’s utility (Daw et al., 2006). In the current model, *E* is calculated for each feature separately:

$$E_{i}=\frac{\max(U)-E\left(U\left(F_{O}\right)\right)}{{\left(1+n_{i}\right)}^{\phi }},$$ 11

where max(*U*) denotes the maximum utility possible irrespective of sampling costs, $E(U(F_{O}))$ will always be less than or equal to max(*U*). *n*_*i*_ denotes the number of previous observations of feature *i*, and ϕ denotes a fixed parameter modulating the influence of *n*_*i*_ on *E*_*i*_. In the case that *F*_*O*_ supports perfect prediction, the comparison of $E(U(F_{O}))$ (i.e., the expected utility, including the costs of sampling each feature given the observed features) to max(*U*) encourages the model to explore when sampling *F*_*O*_ is costly.

Combining the exploration bonus with a softmax choice rule, the probability of sampling feature *m* is:

$$P(F_{m})=\frac{e^{\beta (G(F_{m})+E_{m})}}{\sum_{n}e^{\beta (G(F_{n})+E_{n})}},$$ 12

where β denotes a nonnegative temperature parameter that modulates the stochasticity of the decision process (i.e., *“how often is the feature with highest expected profit chosen?”*). When *E*_*m*_ = 0, and *G*(*F*_*m*_) ≤ 0, the model stops deliberating and commits to a final choice.

### Summary

SEA interleaves concept learning and information sampling, such that they mutually influence one another. Information sampling is akin to a dynamic planning process in which SEA’s concept learning component (i.e., the RMC) serves as an internal model of the environment. For instance, the RMC may learn that red objects tend to be heavy 90% of the time. After observing that an object is red, the RMC would update its expectation that the object is heavy to 90% (Equation 1). Before learning this relationship between color and weight, the RMC would rely on its uninformative prior (50% of objects are heavy, 50% of objects are light) to guide its predictions.

Calculating the probabilities of these unobserved features (e.g., weight) is critical for planning which feature to sample next. The expected utility of a possible state is calculated by combining the probabilities of these states with their utilities (e.g., Equation 7). Importantly, the utility sensitive sampling component does not learn utilities of various states. Instead, SEA is initialized with a utility table (as in Table 1) and with the costs associated with sampling each information source. The conjunction of the concept-learning and utility-sensitive sampling component allows the model to perform active sampling.

Equation 8 is used to calculate the expected utility of states (i.e., specific stimulus feature configurations), abstracting beyond specific actions (or choices). Equation 9 is used to calculated the expected utility associated with sampling an unseen feature, abstracting beyond its possible values. This helps the model to determine if, after sampling a single feature, it should sample another feature. To make this determination, SEA considers information gain (Equation 10) and the exploration bonus for each unsampled stimulus feature (Equation 11) and combines them using a softmax choice rule (Equation 12).

#### Myopic Versus Preposterior Analysis

In deciding which feature to sample, SEA plans ahead for the maximal number of steps, like an adult might when playing a simple game such as tic-tac-toe. In the simulations, we compared SEA to variants that are “myopic” in that they only consider the next step or move (Figure 3). To clarify how the equations interact to support myopic decision making and preposterior analysis, we describe SEA’s behavior in a two-class categorization problem involving stimuli with three binary stimulus features. We assume that the model has already been trained.

Myopic decision making involves simulating the sampling of single unobserved features. Before sampling any stimulus features *F*_*O*_ is [?, ?, ?]. To determine what feature to sample first, the model simulates the effects of sampling each. For instance, the model might calculate the expected utility of the possible states after sampling the first feature (i.e., *F*_*O*_ = [0, ?, ?] or [1, ?, ?]) using Equation 8. The expected utility of sampling this particular feature can be calculated by combining these expected utilities across feature values (Equation 9). The gain in utility from sampling the feature can then be calculated using Equation 10. The exploration bonus for this feature could then be calculated using Equation 11. After performing these calculations for each feature, the decision of what feature to sample would be made using the softmax choice rule (Equation 12).

Conceptually, preposterior analysis is an extension of the myopic algorithm that involves simulation of multiple unobserved features. As in the previous example, the model might begin a trial by calculating the expected utility of the first feature (feature “1”) being “0” (i.e., *F*_*O*_ = [0, ?, ?]). Holding this imaginary feature-value constant, SEA would then simulate the expected utility if other features were subsequently sampled. For instance, to simulate sampling feature “2”, SEA would consider the expected utility of states *F*_*O*_ = [0, 0, ?] and *F*_*O*_ = [0, 1, ?], using Equation 8. It would then abstract over these possible values using Equation 9. It would then calculate the gain in utility and the exploration bonus associated with sampling this feature using Equations 10 and 11.

The process would then be repeated with the value of feature “1” set to 1 (i.e., *F*_*O*_ = [1, ?, ?]). When the depth of the forward search is limited to two steps, the algorithm would commit to sampling a feature after simulating the sampling of two features.^15^ For a three-feature categorization problem, SEA would simulate the sampling of all three features before sampling the first. After sampling one feature, SEA would simulate sampling both remaining features. Importantly, SEA does not learn anything during simulation. The concept learning component is updated only after the final choice is made, and this learning changes behavior on future trials only.

Although the myopic decision algorithm requires minimal computational demands, it lacks the sophisticated behavior that forward search enables (i.e., strategic self-termination and branching). When SEA employs a myopic strategy, it tends to sample more dimensions, and to be less accurate (in terms of its categorization decisions), than when preposterior analysis is employed. These limitations of the myopic algorithm are illustrated in the simulation of experiment performed by Blair et al. (2009; see Strategic Attention Within Individual Trials section).

## Simulations

The proposed model treats decisions as a temporally extended procedure involving sequentially sampling information from the environment, and then committing to a final choice. As reviewed above, these sequential decisions about what features are goal-relevant can influence patterns of eye movements during short-duration decisions (i.e., less than 3 s), which are common in the categorization literature. Whereas successful contemporary theories of categorization tend to rely on attentional parameters that weight stimulus features according to their behavioral relevance, the proposed model can select relevant information through active sampling.

To compare SEA’s behavior to human decision makers and other computational models, we simulate several experiments. In the first, we demonstrate the utility of SEA’s active sampling approach in high-dimensional environments. In the second, we investigate its choice behavior in the classic six problems introduced by Shepard et al. (1961; a well-known test for formal models of categorization). To compare SEA’s sequential sampling behavior to that of human decision makers, we then simulate an eye-tracking experiment conducted by Blair et al. (2009). We then simulate an experiment using the 5/4 category structure (Medin & Schaffer, 1978), and demonstrate that purportedly suboptimal patterns of attention (inferred from both eye-tracking and behavioral choice data) may reflect averaging across individual stimuli associated with distinct scan paths. Finally, we demonstrate that the mnemonic representations resulting from SEA’s active sampling procedure predict human recognition and categorization behavior during the rule-plus exception task (Davis, Love, & Maddox, 2012; Davis, Love, & Preston, 2012b; Love & Gureckis, 2007; Palmeri & Nosofsky, 1995; Sakamoto & Love, 2004).

Rather than fitting the model to each data set, we adopted a conservative approach, and used the same fixed parameters for all simulations (to avoid overfitting). We therefore do not focus on parameter-specific effects in each simulation, but instead consider broader qualitative effects associated with the model architecture.

One-hundred utility units were awarded for correct answers, and no points were awarded for incorrect answers. The exploration parameter, ϕ, was set to 0 (such that the exploration bonus for each feature, *E*_*i*_ was driven purely by potential gain in utility), the decision parameter, β was set to 1, and the cost of sampling each feature, *ℒ_i_*, was set to 10. For the concept learning component, the parameters were set to their default parameters (Anderson, 1991b); the coupling parameter, *c*, was set to 0.3, the α_*j*_ parameter for each label was set to 0.01, and the α_*j*_ parameter for each value of the other features was set to 1.^16^

### Benefits of Selective Sampling

Many real-world learning problems involve identifying a sparse signal hidden within a noisy high-dimensional space (similar to finding a needle in a haystack). Here, we demonstrate the relationship between feature selection and decision accuracy, and demonstrate SEA’s capacity to cope with high-dimensional problems. In particular, we illustrate how selective sampling can increase learning efficiency by comparing the learning performance of a model that actively samples information to a model that samples every feature on every trial. The purpose of this simulation is not to demonstrate that the model performs similarly to human decision makers in high-dimensional environments (we are unaware of category-learning data sets with similar structure). Rather our goal is to demonstrate how selective sampling in SEA can facilitate learning.

**Figure 4 fig4:**
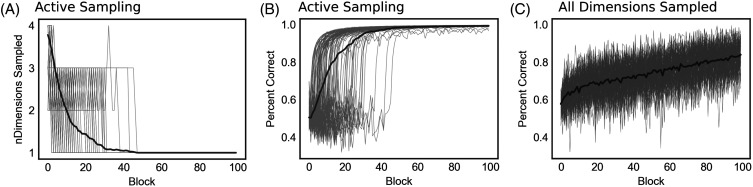
Benefits of Selective Sampling *Note*. In the active-sampling model, the number of features sampled (A) negatively covaried with the slope of the learning curve (B). This reflects the models ability to efficiently explore unsampled features (i.e., by considering the number of times each feature has been observed; Equation 11), and capitalize on the single reliable feature in the simulation environment. The selective sampling model (B) learned more quickly than a comparable model in which all stimulus features were always sampled (C).

In both simulations, the stimuli were composed of 99 random binary features (generated through a process similar to coin flipping), and a single binary feature that perfectly correlated with the category label. During each of the 100 repetitions of each simulation, SEA was trained over 100 blocks of 10 trials. As full preposterior analysis (exhaustive forward search through the 100-dimensional space) imposes high computational demands, SEA was set to use a “myopic” strategy in which forward search was limited to one step ahead. In our first simulation (Figure 4A and 4B), the cost associated with sampling each feature limited SEA’s sampling behavior to five or fewer features per trial. Nevertheless, SEA quickly found the signal feature, and learned to ignore the others.[Fig fig4]

In the second simulation (Figure 4), instead of performing active sampling, SEA sampled every feature on every trial. The comparison between the two models demonstrates how selective sampling based on expected utility can improve learning. Real-world environments typically impose some cost for information sampling (e.g., time, effort, or monetary), and the number of features considered during deliberation is a function of these costs. Therefore, learning efficiency can be impaired when a models inductive bias (or prior) is inappropriately matched to the environment. Specifically, when estimated costs are too low, a decision maker may sample too many features. Conversely, when costs are too high, the decision maker may fail to efficiently explore the domain, and develop inaccurate beliefs as a result. A strongly cost-sensitive decision maker, for example, may identify a single weakly informative stimulus feature, and subsequently fail to identify more-reliable decision strategies (i.e., using a different stimulus feature, or considering a set of features).

### Shepard et al. (1961)

**Figure 5 fig5:**
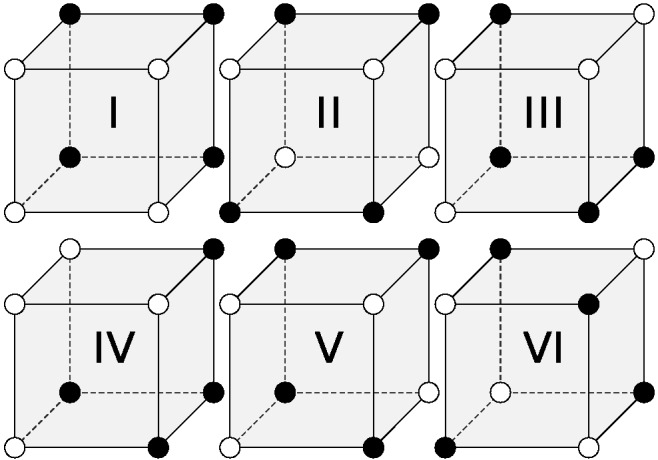
Geometric Depiction of the Six Problem Types (Shepard et al., 1961) *Note*. Members of each category are denoted by white and black spheres. In the Type I problem, a single dimension is relevant. In the Type II problem, two dimensions are relevant, and decisions makers must employ a logical XOR rule. In the Type III, IV, and V problems, all dimensions are informative, but the categorization structures differ in interesting ways. In the Type VI problem, all stimulus dimensions must be considered.

For decades, the six problems developed by Shepard et al. (1961) have been a benchmark for testing formal categorization theories. In Shepard’s six problems, stimuli consisting of three binary features, are used to define six different category problems (Figure 5). The Type I problem is a one-dimensional task in which a single dimension is relevant and the other dimensions can be ignored. The Type II problem is a two-dimensional rule-based task requiring the learner to employ a disjunctive exclusive-or (XOR) rule. In the Type III, IV, and V problems, all dimensions are informative, and for each category, all but one member can be categorized according to the same strategy, while the remainder must be categorized using a different strategy. These three problems also differ in interesting ways; the Type III problem, for instance, can be solved using two dimensions,^17^ and the Type IV problem is characterized by a linearly separable prototype structure. Finally, in the Type VI problem, all three dimensions are relevant, and participants must essentially memorize the individual stimuli.[Fig fig5]

**Figure 6 fig6:**
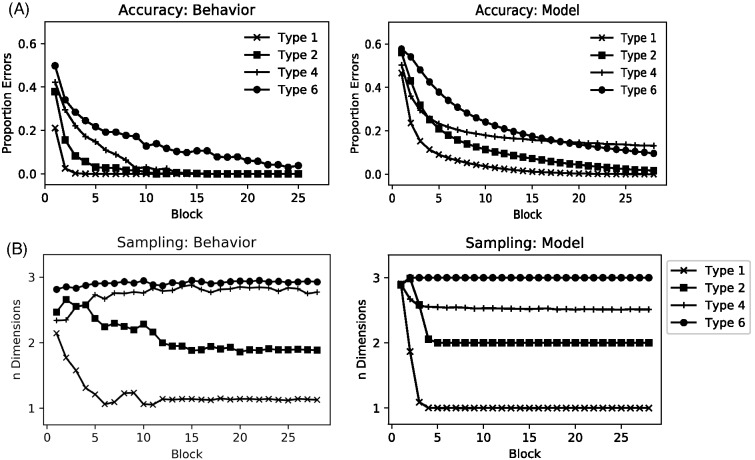
Human and Model Accuracy and Sampling Behavior for the Six Problems Described by Shepard et al. (1961) *Note*. (A) Left: Human categorization accuracy by learning block. Right: Model Accuracy by learning block. Mirroring behavior of the Rational Model of Categorization (RMC; Anderson, 1991b), learning of the Type IV was attenuated during later blocks relative to other problem Types. For discussion of this effect, please see the original text. (B) Left: In an eye-tracking study, Rehder and Hoffman (2005a) found that human participants learned to selectively fixate on behaviorally relevant stimulus dimensions across blocks. Right: Like human decision makers, the model learned to selectively sample from behaviorally relevant stimulus dimensions. Vertical-axis: number of dimensions sampled. Horizontal-axis: learning block.

A typical finding, which has been both replicated (Nosofsky et al., 1994) and extended (Feldman, 2000; Love, 2002; Nosofsky & Palmeri, 1996), is that the initial difficulty of each task (as measured by the proportion of choice errors) closely reflects the number of dimensions that must be considered (Medin & Schaffer, 1978; Nosofsky, 1984; Shepard et al., 1961). Therefore, the tasks tend to increase in initial difficulty from Type I to II, from II to {III, IV, V}^18^, and from {III, IV, V} to VI (Figure 6A, left). In support of this idea, several formal categorization models that include selective attention, are able to closely predict this behavioral effect (Nosofsky et al., 1994), while Bayesian models (like Anderson’s Rational model; Anderson, 1991b) tend to underestimate differences between the problems during early learning (Nosofsky et al., 1994).[Fig fig6]

We simulated the experiment performed by Rehder and Hoffman (2005a) who used eye tracking to investigate whether the differences in behavioral accuracy between rules might reflect differences in information sampling. Their important finding was that, following learning, eye movements closely reflected the behavioral relevance of each dimension (Figure 6B, left). These results provide compelling support for the idea that differences in attentional strategies underlie observed differences in behavioral accuracy between rules. The results also indicate that attention operates, not only at later decisional stages but also can influence information sampling behavior.

We performed 1,000 simulations of this experiment using full preposterior forward search. Each simulation involved 28 learning blocks. In each block, each stimulus was presented in random order. Although we simulated all problems, our goal was to compare our findings to those of Rehder and Hoffman (2005a), so we report only results associated with problems I, II, IV, and VI.^19^

The model predicted the correct ordering of six problem difficulties (Figure 6A, right). Perhaps more interestingly, the model learned to selectively sample behaviorally relevant stimulus dimensions across learning blocks (Figure 6B, right). Although the model used the same parameters for this simulation as for all others, this sampling behavior resembled that of human decision makers. One interesting difference, however, is that while humans tended to sample from all three dimensions during performance of the Type IV problem, the model sampled an average of 2.5 dimensions. This reflects the prototype structure of this problem, which allowed SEA to strategically self-terminate on roughly half of the trials. The results of Rehder and Hoffman (2005a) imply that only a small percentage of high-performing participants may have self-terminated in this *stimulus-specific* way. Although it is potentially interesting that the model identified this efficient sampling strategy, the propensity for self-termination will correlate with information cost.^20^ In the next section, we apply SEA to a study whose design is ideal for evaluating whether people self-terminate and branch in a stimulus-specific way.

### Strategic Attention Within Individual Trials

The results from the simulations of the Shepard et al. (1961) and Rehder and Hoffman (2005a) experiments demonstrate that the model is capable of mirroring the human tendency to strategically sample behaviorally relevant information based on learned category structure. This type of *feature-based* attention is important for improving the efficiency with which decisions can be made. In many contexts, however, decision makers can further reduce the amount of information sampled by considering *stimulus-specific* factors.

For instance, in the category structure shown in Table 2 (and in Figure 1B), the “indicator” dimension (D1), by itself, is not predictive of category membership, but determines which of the two remaining dimensions should be sampled. When D1 = 1, for instance, the decision maker should sample D2 next, but when D1 = 2, only D3 is informative. Thus, 100% accuracy can be achieved by first sampling the indicator dimension, and then strategically sampling only one of the remaining dimensions.^21^ [Table tbl2]

Participants in Blair et al’s study learned to perform the task through trial-and-error, until either reaching a learning criterion of 24 correct consecutive trials or until a maximum of 200 total trials. Participants then performed an additional 72 (“transfer”) trials of the same stimuli without feedback. Data from participants (42%) who did not reach the accuracy criterion were excluded from the primary analyses. The findings indicated that participants were able to employ stimulus-specific attention during information sampling. Participants tended to selectively sample dimensions 2 and 3 depending on the value of dimension 1, and therefore, spent more time fixating on dimensions 1 and 2 for stimuli belonging to category A or B, and more time fixating on dimensions 1 and 3 for those belonging to category C or D.

To isolate behavioral effects reflecting the depth of the forward search process, we simulated this experiment using two model variants. The standard SEA model included stimulus specific attention and exhaustive preposterior search, while the myopic model considered only one step into the future. As each dimension is equally predictive in isolation, the myopic model was no more likely to sampling the indicator dimension than a nonindicator dimension. The myopic model, therefore, should sample the indicator dimension first on roughly one third of trials. In these trials, it could then select the appropriate nonindicator dimension to sample. However, if a nonindicator dimension was sampled first, the model should then randomly sample either the indicator dimension (and then self-terminate) or the other nonindicator dimension (and then sample the remaining dimension). As a result, when using a myopic strategy, the model should tend to sample a greater number of stimulus features than when preposterior analysis is used.

After reaching the learning criterion, the standard model correctly classified 93.3% of the remaining 72 transfer items. Mirroring human sampling behavior, the standard model tended to sample all stimulus dimensions early in learning, but then tended to sample only two dimensions per trial: first D1, and then either D2 or D3. (The features sampled were optimal on 98.6% of trials.) After reaching the learning criterion, the myopic model correctly classified 67.9% of the remaining 72 items. Like the standard model, the myopic model tended to sample all dimensions during early learning, and sample fewer dimensions later in learning. However, demonstrating the benefits of planning during information sampling, the myopic model tended to sample more dimensions than the standard model after learning (*M* = 2.33 instead of *M* = 2).

### Modeling Eye Movements in the “5/4” Categorization Task

The previous results from Blair et al. (2009) were inconsistent with a standard view of selective attention, but were compatible with SEA’s sampling account which can lead to different sampling patterns for different stimuli. One possibility is that classic studies consistent with selective attention accounts in part reflect the averaging of different sampling patterns for different stimuli. In this section, we consider this possibility by revisiting Medin and Schaffer’s (1978) “5/4” categorization structure (shown in Table 3), which was originally used to differentiate prototype- and exemplar-model accounts of category representation.[Table tbl3]

During a training phase, participants typically learn to categorize the first nine stimuli (A1–A5 and B1–B4) through trial and error. In a subsequent transfer phase, the participants also categorize the seven transfer items (T1–T7). The task is somewhat ill-defined, in that no single feature perfectly predicts the category label. Instead, the categories have a prototype structure (category A: [0, 0, 0, 0]; category B: [1, 1, 1, 1]), and the features differ in terms of how reliable they are with regards to prediction of the correct response. As shown in Table 3, the “High1” and “High2” features each correctly predict the category label for seven of the nine training items, the “Med.” feature predicts the correct category label for six out of the nine training items, and the last feature (“Low”) predicts only five of the training items correctly.

Viewed through the lens of categorization models that include feature-wide attention parameters, an optimal decision maker should place no-weight on the least-informative feature. Exemplar models (e.g., Nosofsky, 1986), but not prototype models (Minda & Smith, 2002; Nosofsky, 1987), indicate that human participants tend to assign substantial weight to this feature. This seemingly suboptimal pattern of attentional weighting has been interpreted as evidence favoring the prototype account of category representation (Minda & Smith, 2002).

To independently assess the attention devoted to each feature, Rehder and Hoffman (2005b) used eye tracking to measure fixations to each feature across training. Visual features were randomly assigned to each category feature (i.e., the features shown in Table 3) in a counterbalanced fashion (across participants) to account for effects associated with visual salience. Through trial-and-error, participants trained until either completing 21 training blocks in total, or completing two consecutive blocks without error. Each block involved a single presentation of each of the nine stimuli in random order. During the subsequent transfer phase, participants categorized all 16 stimuli in each of two blocks. Each transfer block consisted of a single presentation of each stimulus in random order, and no feedback was presented. Matching the predictions of exemplar theory, a key finding was that the majority of participants actually do display this seemingly suboptimal attentional pattern.

In SEA, optimality is defined with respect to the maximization of expected utility. From this perspective, an active-sampling learner should seek to optimize scan paths for individual stimuli (i.e., minimizing sampling costs and maximizing reward). One possibility is that the attentional pattern observed by Rehder and Hoffman (2005b) might reflect an average across different optimal scan paths for individual stimuli.

**Figure 7 fig7:**
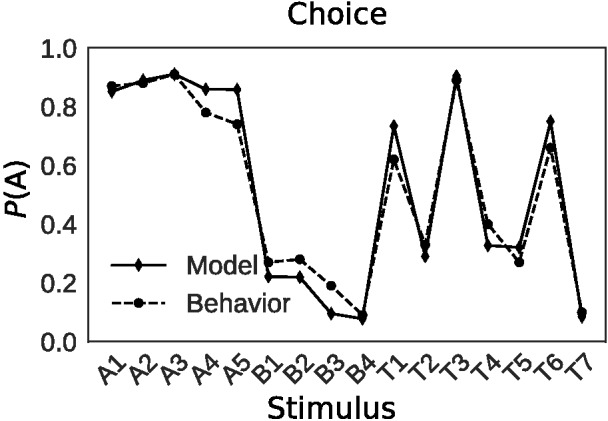
5/4 Categorization Task: Comparison of Human and Model Categorization Behaviour *Note*. Human data from (Rehder & Hoffman, 2005b).

To investigate this possibility, we simulated this experiment 1,000 times. Although the same parameters were used for all simulations, SEA’s choice behavior closely resembled that of human decision makers (Pearson *r* = 0.98; Figure 7; Rehder & Hoffman, 2005b). SEA’s sampling behavior also resembled human eye-movement data. Human decision makers were more likely to sample the highly informative features [*M*(High1) = 80%, *M*(High2) = 80%] than the moderately informative feature (*M* = 75%), and were more likely to sample the moderately informative feature than the least informative feature (*M* = 60%). SEA displayed the same ordering of feature fixation probabilities (High1 = 83%, High2 = 83%, Med. = 66%, Low = 17%).[Fig fig7]

These results provide a new vantage point on optimality for this task. From the perspective of models that have feature-wide attention, it is suboptimal to place any weight (i.e., sample) the least informative feature in the 5/4 problem. However, according to SEA, featureal relevancy is contingent on what information has previously been sampled. According to SEA, depending on the stimulus and scan path, the so-called least informative feature can be highly informative. In those cases, SEA will sample this feature to maximize utility. SEA’s strategic sampling leads it to sample 2.42 features on average for the 5/4 problem whereas a feature-wide attention model would need to consider 3.0 features on every trial. In light of this result, one conclusion is to exercise caution in characterizing attentional allocation as suboptimal when stimulus-specific scan paths can increase sampling efficiency.

### Rule-Plus Exception

In the previous simulations, we focused on eye-tracking studies as they provide an independent estimate of attention (assuming a typically strong coupling between eye movements and attention holds, e.g., Deubel & Schneider, 1996). However, we intend our theory and model to not only accurately predict human sampling behavior but to additionally account for effects thought to reflect the resulting mnemonic structure. To illustrate how our model performs subsequent recognition memory, we applied SEA to an experiment using the rule-plus exception category structure (Table 4). In this structure, most stimuli can be accurately sorted into categories according to a simple rule, but the remaining exception items must be recognized, and categorized according to a different strategy. Behavior on this task reveals interesting differences in how rule-following and exception items are encoded.[Table tbl4]

Although rule-following items tend to be more easily learned (as estimated by categorization accuracy), exception items tend to be better recognized (as estimated by subsequent old–new recognition-test accuracy; Davis, Love, & Maddox, 2012; Davis, Love, & Preston, 2012b; Love & Gureckis, 2007; Palmeri & Nosofsky, 1995; Sakamoto & Love, 2004). This is thought to reflect stronger encoding of the rule-irrelevant features for the exception items. Differences in categorization and recognition accuracy between the rule-following and exception items, therefore, suggest differences in the organization of conceptual knowledge. As single-system categorization models (e.g., Nosofsky, 1986) have difficulty accounting for this effect, dual-process frameworks (involving separate representational systems for rule-following and exception items) have been proposed (e.g., Nosofsky et al., 1994). We predicted that due to its minimization of sampling cost, SEA would develop incomplete representations of rule-following items (i.e., ignoring rule-irrelevant features), and would sample more features for exception items (as these need to be differentiated from rule-following items, and then sorted according to a different strategy).

In SEA, recognition strength is modeled (by the concept-learning component) as the likelihood of the observed stimuli, given the learned clusters:

$$\text{Recognition Strength}=\sum_{k}P(F_{O}|k)P(k),$$ 13

where *P*(*k*) denotes the prior probability of existing clusters (Equation 4). Our estimate of recognition strength therefore reflects the degree to which a stimulus “activates” the existing clusters. This variable comprises the denominator in Equation 3 and so plays an important role in normalizing estimates of *P*(*k*|*F*_*O*_).

**Figure 8 fig8:**
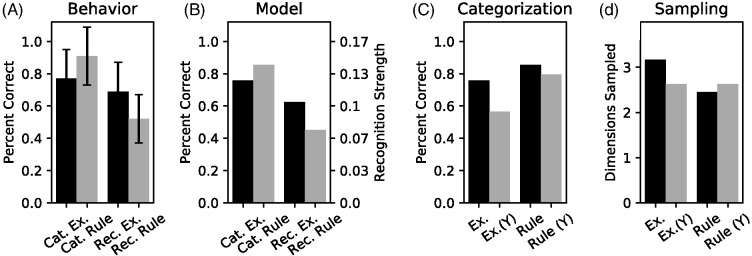
Rule Plus Exception Results *Note*. (A) Human Behavior: Recognition (“Rec.”) and categorization (“Cat.”) accuracy for rule-following (“Rule”) versus exception (“Ex.”) stimuli (Davis, Love, & Preston, 2012a). Although categorization accuracy was greater for rule-following items, recognition accuracy was greater for exception items. Error bars reflect 95% confidence intervals. (B) Model Behavior. Mirroring human behavior, the model displayed greater categorization accuracy for rule-following items than for exception items, but greater recognition strength (Equation 13) for the exception items than the rule-following items. (Note differences in the vertical-axis scale for categorization accuracy and recognition strength). (C) Model Categorization Behavior: Categorization accuracy for rule and exception items for the standard and yoked (“Y”) models. Mirroring human behavior, both models displayed better categorization accuracy for rule-following items than for the exception items. Accuracy for the yoked model was lower than that of the standard model. Model-type and stimulus-type interacted such that the yoked model displayed a greater difference in accuracy between rule-following and exception stimuli than the standard model. (D) Model Sampling Behavior. Although the standard model sampled a greater number of features for exception items than for rule-following items, the yoked model did not.

The model results conformed to the pattern of human results as rule-following items had an accuracy advantage during learning, and a disadvantage during subsequent recognition test (Figure 8). As shown by the black bars in Figure 8D, our model sampled fewer features (*M* = 2.45) for rule-following items than for exception items (*M* = 3.17) during learning, reflecting a learned strategy of sampling until the presence or absence of an exception item could be determined.[Fig fig8]

To better understand the consequences of this learned sampling strategy, we simulated a “yoked” model that inherited the scan paths from a simulation of our standard model. This removes the possibility of hypothesis-dependent sampling, as the concept-learning and active-sampling components were decoupled.^22^ Compared to our regular model, the yoked model’s performance was particularly impaired on the exception items. The yoked model made disproportionately more errors to the exception items during learning (see Figure 8C) and was worse than our standard model in recognizing these items. This finding illustrates the importance of strategic sampling in this task. In particular, the categorization task necessitated greater sampling of information about exception items, leading to a more complete representation of these items in memory.

## General Discussion

SEA describes how people strategically sample information while learning and making decisions. It consists of a Bayesian learning component, which models beliefs about the world, and an information-utility component that conducts a goal-directed forward search based upon these beliefs. Interactions between the two components allow the model to actively learn about the external world by sequentially sampling from information sources expected to provide useful information. In SEA, usefulness reflects both a drive to maximize expected gain in utility (exploitation of existing knowledge), and a drive to maximize knowledge of the external world (exploration). As a consequence of active sampling, SEA’s knowledge of the world reflects the utility function it strives to optimize (Table 1 provides an example of a utility function that would maximize decision accuracy). Although SEA differs from “single-step” categorization models that sidestep questions related to active sampling, and instead contort representations of encoded dimensions based on their behavioral relevance (e.g., Kruschke, 1992; Love et al., 2004; Nosofsky, 1986), its active sampling behavior leads to the development of dense representations along dimensions expected to be behaviorally relevant, and sparse representations along dimensions expected to be irrelevant. As a result, SEA provides a compelling account for many aspects of human categorization behavior. Interestingly, although each component can be considered normative in its own right, as a consequence of the recurrent interactions between learning and information sampling, SEA can develop and maintain beliefs that systematically deviate from reality (Figure 2).

As our goal was to offer a general theory of how attentional-like behavior could emerge from sequentially sampling information according to its expected utility (Trommershäuser et al., 2006), SEA was not tuned to any of the individual tasks. The default parameters from the RMC were used throughout. Similarly for each simulation, an arbitrary cost of 10 utility units was imposed for sampling each stimulus feature. Although it was not tuned to particular data sets or tasks, SEA was able to capture a wide range of category learning findings. For example, in addition to capturing the basic difficulty ordering of the six problem types described by Shepard et al. (1961), SEA correctly captured known human sampling behavior during category learning (Figure 6). Thus, by combining the Bayesian concept-learning component with a utility-sensitive sampling component, SEA is able to account for effects that were thought to require a dedicated postencoding attention-weighting mechanism (Kruschke, 1992; Nosofsky et al., 1994).

SEA can additionally address sampling phenomena that are outside the scope of existing models with attentional mechanisms. Rather than initially encoding all information used to form the decision, and then contorting these encoded representations based on their behavioral relevance, SEA’s information-utility component allows the model to select relevant information through a dynamic forward search process. This allows the model to allocate attention flexibly within individual trials (as in Blair et al., 2009). This ability is unavailable to models in which attention operates at the level of individual features, but not at the level of individual stimuli. Moreover, as “indicator” features (such as those in the category structure considered by Blair et al., 2009, which indicate the next appropriate feature to sample) can be considered as contextual cues signaling appropriate decisional strategies, SEA’s active sampling procedure naturally accounts for effects associated with context-gated knowledge partitioning (Lewandowsky et al., 2006; Little & Lewandowsky, 2009; Yang & Lewandowsky, 2003).

This capacity to flexibly sample from information sources expected to provide useful information supports an alternative interpretation of findings associated with the classic 5/4 category structure (Medin & Schaffer, 1978; Rehder & Hoffman, 2005b). In our simulations, SEA sampled stimulus features at an overall rate consistent with the best-fit attention weights from the Generalized Context Model (GCM; Nosofsky et al., 1994). However, these overall sampling proportions arose in SEA from averaging heterogeneous sampling patterns across the individual stimuli. One possibility is that heterogeneous sampling behavior might also explain why these weights arose for the GCM. By characterizing each category decision as the culmination of an active sampling process, SEA offers a rich account of the microstructure of each trial that can be tested experimentally. In cases such as the 5/4 studies, this disaggregation offers novel insights into classic categorization tasks and alternative accounts of the behavioral results.

Although we have focused on eye-tracking studies, we intend SEA to apply to other kinds of behavior. Even in cases in which all stimulus features fall within the same spatial location, its selective sampling processes should still be operable. To illustrate this point, we applied SEA to a rule-plus-exception category learning problem in which most items followed a simple rule, and the remaining exception items had to be categorized according to a different strategy (Davis, Love, & Maddox, 2012; Davis, Love, & Preston, 2012b). Like human decision makers, SEA made more errors on exception items during learning while also showing enhanced recognition for these items following learning. This reflects increased representational density for the rule-following items relative to the exception items; as SEA tended to sample more stimulus features for exception items (sampling each stimulus until it could be determined whether it was an exception).

We also aimed to show how behaviors beyond the scope of classic models of selective attention could be explained in terms of strategic sampling. That SEA could capture the qualitative data patterns in these studies, and in cases offer novel interpretations and predictions, is a strength of this work. In this contribution, we did not stress comparison to alternative models, though we did note where classic selective attention models and myopic versions of SEA that do not perform full-look-ahead search would fail. In the future, finer-grained model comparisons and fits to data, including to individual participants, can assist in evaluating alternative strategic sampling models.

By considering information gathering to be an integral component of category learning, the current approach recasts categorization as a dynamic decision-making problem. This is similar to a model-based reinforcement-learning approaches in which the learning agent incrementally builds a model of the external world, while at the same time using the model to adjust its policy (i.e., guide the agent’s choices; Sutton, 1990, 1991). In these kinds of dynamic decision-making tasks, optimal performance requires a delicate balance between exploration (which provides the highest returns according to current estimates of utility) and exploitation (which help the agent to discover options with potentially greater utility; Kaelbling et al., 1996; Sutton & Barto, 1998).

Although it places less emphasis on managing the exploration/exploitation trade-off, the model most similar to SEA is likely that developed by Nelson and Cottrell (2007). This model combines a Bayesian concept-learning component (which was designed for the six problem types described by Shepard et al., 1961), with an active sampling procedure driven by the expected *information gain* of each stimulus feature (Nelson, 2005). In conjunction with a fixed cost for sampling each feature, the sampling procedure encouraged the model to selectively sample task-relevant stimulus features; mirroring previously observed patterns of human eye movements (Rehder & Hoffman, 2005a). SEA is additionally able to predict the correct ordering of problem difficulties (i.e., Type I < II < IV < VI),^23^ a common touchstone for evaluating formal categorization models. Nelson and Cottrell’s work can be seen as a special case of SEA. First, SEA includes a flexible concept-learning component that is capable of learning a wide variety categorization problems. Second, it is flexible, in that it can maximize accuracy or expected utility. This is useful when the maximization of utility and accuracy represent distinct objective functions.

Below, we discuss some possible elaborations of SEA after first considering some implications of this work.

### Bayesian Discriminative Learning

One popular distinction in machine learning is between discriminative and generative models (Ng & Jordan, 2002). In brief, generative and discriminative models characterize the task of the learner differently. Generative models attempt to learn an internal model of each class (i.e., category). In contrast, discriminative models attempt to find a boundary that separates classes. Generative models are typically Bayesian in form, whereas discriminative models include decision trees, Support Vector Machines, regression approaches, and some (but not all) connectionist models. In generative models, the learning task is to estimate the joint probabilities between all variables. These models assume that a hidden or latent variable (e.g., a category label) generates the observed features. In contrast, discriminative models perform a conditional estimation. For example, logistic regression only estimates the probability of a class (i.e., category) as a function of the predictive features. In this sense, discriminative models are more focused by the task, whereas generative models address a broader estimation problem, though models of all types have an inductive bias to make learning tractable.

SEA displays characteristics of both generative and discriminative models. SEA is a generative model in that it builds an internal model of the world that can be sampled from. On the other hand, its internal model is heavily biased by the discriminative pressures of the tasks it performs, which results from its utility-driven sampling. Therefore, rather than attempting to build an unbiased model of the world, SEA samples information that it expects will be useful for performance of the task. Although learning to classify items as members of one of two contrasting categories, for example, SEA will naturally focus on information that discriminates the two categories. SEA is therefore a generative model whose internal model of the world is shaped by its goals. This is also the defining characteristic of the SUSTAIN clustering model (Love, 2005). In effect, SEA follows SUSTAIN’s basic principles, but updates and places these principles within a Bayesian framework. This allows SEA to handle uncertainty, display strategic sampling behavior, and model changes in tasks and goals via changes of its utility function.

These characteristics allow SEA to capture behaviors not typically associated with Bayesian models. One such behavior is blocking (Kamin, 1969) in which knowledge of an informative stimulus feature can interfere with the learning of another. For example, consider a trial-by-trial category learning task in which the shape feature is predictive of category membership, and all other stimulus features are irrelevant. During early trials, SEA predicts that all features will be sampled occasionally, consistent with the findings of a uniform prior in repeated resource allocation games (Benartzi & Thaler, 2001; Langholtz et al., 1997). However, assuming some cost to sampling information (e.g., a desire to minimize cognitive effort), eventually, only shape information will be sampled and other stimulus features will be ignored. Thus, if another feature that was previously behaviorally irrelevant becomes informative, SEA would be unlikely to learn this new relationship.

The simulations reported here involved classification learning in which the learner aims to predict the category label from the features. In this induction task, all the features are known and the category label is inferred. However, other induction tasks are possible, such as inference learning, in which the learner knows the category label and one of the features is inferred instead (e.g., *This is a fish. Does it have scales?*). Although inference and classification learning are informationally equivalent (after feedback is provided, inference and classification learning provide the same information to the learner), but strongly influence what human decision makers ultimately learn (Chin-Parker & Ross, 2002; Rehder et al., 2009; Sakamoto & Love, 2010; Yamauchi et al., 2002; Yamauchi & Markman, 1998). In classification learning, people tend to learn information that discriminates between the two categories. In inference learning, however, people tend to learn more about the internal structure of each category.

When its utility function is adjusted to reflect each task, SEA’s behavior is consistent with these results. For classification learning, SEA’s utility function should emphasize predicting the category label. In inference learning, SEA’s utility function should emphasize predicting whatever feature is absent on the current trial. In effect, the task demands should shape SEA’s utility function, which will in turn shape SEA’s internal model; consistent with the psychological theory of how human memory is shaped by these tasks (Markman & Ross, 2003). Related manipulations that alter the presentation order of features and label (Ramscar et al., 2010) or the isolation of categories (Goldstone, 1996) could also be accommodated in a principled way by tailoring SEA’s utility function.

### Future Directions

One line of future work is improving SEA’s basic components. For example, SEA’s learning component relies on Anderson’s (1991b; Anderson & Matessa, 1990) RMC. Virtually any other concept learning model could be used that can perform forward planning by estimating the probabilities of unobserved features. Basic improvements could also be made to SEA’s information-utility component. SEA’s information-utility component performs an exhaustive forward search; evaluating the full breadth and depth of the decision tree defined by the stimulus attributes. As the number of possible branches increases exponentially with the number of features considered, this exhaustive approach is prohibitively expensive for all but the simplest problems.

We did consider a myopic version of SEA that lowered search costs (in terms of computation) and performed well in some environments (Figure 4). On occasions, people may also engage in simple myopic search strategies (e.g., Busemeyer & Rapoport, 1998). However, our results also make clear that people can also engage in more sophisticated search strategies. One possibility would be to allow the model itself to determine the appropriate depth of the forward search (Snider et al., 2015). For the problems considered here, searching only two steps ahead would have been sufficient to support human-like behavior. A model starting with a shallow search and then progressing deeper until reaching a performance plateau could be a viable model for human information sampling. More sophisticated search procedures, of course, could also improve computational efficiency, and/or better capture human characteristics. For example, Google DeepMind’s AlphaGo, which defeated a champion human Go player, relies on Monte Carlo tree search to selectively explore the most promising parts of the search space (Silver et al., 2016). People may similarly rely on memory to retrieve the most effective search strategies used in the past (Logan, 1988).

In addition to considering the search strategy, where the primary concerns are computational complexity and pruning of the search space, consideration of alternative evaluation strategies, such as a “confirmatory” or “positive” testing strategies (Klayman, 1995), would also be fruitful. In a sense, SEA already explains people’s tendency to engage in confirmatory behavior in terms of using a biased internal model for forward planning. Therefore, SEA can be considered an alternative account of how confirmatory behavior can arise. Rather than solely reflecting a faulty reasoning process, confirmatory behavior may reflect a biased internal model. In other words, a decision maker could try to reduce uncertainty through preposterior analysis, but fail to make accurate predictions as a result of inaccurate beliefs. SEA’s capacity for this behavior makes it susceptible to phenomena like blocking. Our hope is that it will advance our understanding of broader phenomena, such as echo chambers or filter bubbles, in which personalized information searches and social media use can lead to underexposure to alternative viewpoints, resulting in inaccurate, or incomplete, worldviews (Pariser, 2011).

Incorporating alternative search and evaluation strategies may increase SEA’s quantitative fit to human data, as could incorporating additional noise sources into SEA’s evaluation and decision processes. Across the simulations considered here, SEA’s behavior could be characterized as somewhat idealized in comparison to human participants. In this contribution, we prioritized illustrating basic principles and performance patterns over quantitative fit. Future efforts may emphasize quantitative fit and measurement of individual differences in learning and information sampling strategies.

SEA also suggests fruitful research avenues to explore in psychology and neuroscience. Traditional models of selective attention (e.g., Kruschke, 1992; Love et al., 2004; Nosofsky, 1986) have been useful at both the behavioral and neural levels of analysis (Braunlich & Love, 2019; Mack et al., 2013). Although these models provide a principled way to investigate how information is organized during decision making, they tend to sidestep questions related to active sampling processes which unfold across time during deliberation. SEA, however, posits that more sophisticated sampling processes unfold across time during deliberation. This opens a number of avenues for future investigation. For example, SEA proposes that an optimal decision maker should consider the expected gain in utility from each feature, *G*(*F*_*i*_), Equation 10. This variable can be subdivided into three subcomponents: The expected cost of sampling a feature, the expected gain in utility of sampling the feature without consideration of its cost, and the expected reliability (i.e., expected inverse variance) of each feature. SEA also provides an estimate of the expected reduction in uncertainty about the appropriate final choice from sampling each feature. In addition to the issues outlined above regarding search strategies, each of these variables may be of interest to scientists interested in examining intratrial attentional effects.

## Conclusions

Current models of categorization provide a compelling account for how information is organized to support advantageous decision making (Figure 1A). Although these models provide important insights into decision-making strategy, they sidestep the sequential and contingent information sampling processes that occur within individual trials (Figure 1B), and which are necessitated by the computational demands of interacting with the high-dimensional real world. As shown in Figure 4C, encoding all available sensory information in high-dimensional environments is computationally inefficient (at least at high resolution; Goffaux et al., 2010). Accordingly, decision makers appear to draw on their existing knowledge to selectively sample information from sources expected to provide behaviorally relevant information (Blair et al., 2009; Najemnik & Geisler, 2005; Nelson & Cottrell, 2007; Yang et al., 2016). Active-sampling learners therefore can develop beliefs about the world that systematically deviate from reality (Figure 2), particularly when competing demands for exploration and exploitation are not balanced appropriately.

SEA describes these bidirectional effects on the development of conceptual knowledge. Although it is largely formulated at the computational level (Marr, 1982), it makes predictions about how behavior should unfold both within and across trials, and should be useful in understanding and predicting human behavior. SEA should serve as a useful guide for understanding attentional effects, learning, and decision phenomena underlying the development of biased representations of the external world. Such effects are not only common in the laboratory, but are of fundamental importance for understanding how capacity-limited decision makers interact with the external world.

## Supplementary Material

10.1037/rev0000287.supp

## Figures and Tables

**Table 1 tbl1:** Example Utility Table

State	*a*_*p*_	*a*_*q*_
*s*_*p*_	100	0
*s*_*q*_	0	100

**Table 2 tbl2:** Category Structure Used by Blair et al. (2009)

Category	D1	D2	D3
A	1	1	1
A	1	1	2
B	1	2	1
B	1	2	2
C	2	1	1
C	2	2	1
D	2	1	2
D	2	2	2
*Note*. While eye-tracking data were collected, participants learned to sort the eight stimuli into four different categories (A–D). The A and B categories shared the same relevant dimensions (D1 and D2), as did categories C and D (D1 and D3). The optimal strategy was to first sample Dimension 1 (D1), and then sample either D2 or D3 depending on its value (i.e., if D1 = 0, the optimal strategy would be to sample D2, otherwise, one should sample D3). See also Figure 1B.			

**Table 3 tbl3:** The 5/4 Category Structure (Medin & Schaffer, 1978)

Stimulus	High1	High2	Med.	Low
A1	2	2	1	2
A2	2	2	1	1
A3	2	2	2	1
A4	2	1	2	2
A5	1	2	2	2
B1	2	1	1	2
B2	1	2	1	2
B3	1	1	2	1
B4	1	1	1	1
T1	2	1	2	1
T2	2	1	1	1
T3	2	2	2	2
T4	1	2	1	1
T5	1	1	2	2
T6	1	2	2	1
T7	1	1	1	2
*Note*. Med. = medium.				

**Table 4 tbl4:** Rule-Plus Exception Category Structure (Davis, Love, & Preston, 2012b)

Category	Item type	D1	D2	D3	D4
A	Train*	2	2	2	2
A	Train	1	1	1	2
A	Train	1	1	2	1
A	Train	1	2	1	1
B	Train*	1	2	2	2
B	Train	2	1	1	2
B	Train	2	1	2	1
B	Train	2	2	1	1
—	Test	1	1	1	1
—	Test	1	1	2	2
—	Test	1	2	1	2
—	Test	1	2	2	1
—	Test	2	1	1	1
—	Test	2	1	2	2
—	Test	2	2	1	2
—	Test	2	2	2	1
*Note*. Participants in this study learned to categorize based on the first eight stimuli (Item Type: “Train”). By attending only to the first feature (“D1”), participants would be able to categorize three of the four stimuli within each category. Exception items (marked with an asterisk) violate this simple rule, and thus require attention to other features. After category training, item recognition for the rule-following and exception items is compared via a two-alternative forced choice task, involving comparison to eight additional test items (Item Type: “Test”).					

**Figure B1 fig9:**
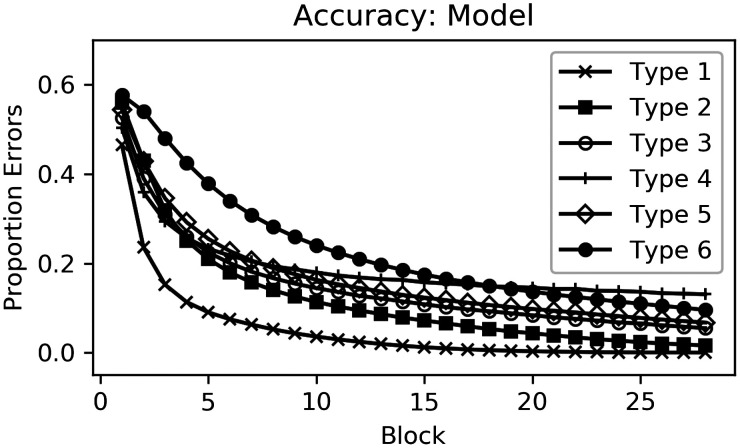
Simulation of Shepard et al. (1961): All Rules

## Footnotes

1 In the category-learning literature, stimuli with two-four features are common (e.g., Nosofsky, 1986; Shepard et al., 1961), and stimuli with 16 features are considered to be high-dimensional (e.g., Vong et al., 2019).

2 For example, when making a diagnosis a doctor might initially conduct a blood test, and then choose an additional test once the results are known.

3 Throughout the article, we use the terms “information sources”, “features”, and “dimensions” interchangeably. “Information source” has a more general connotation than stimulus “dimensions” or “features.” “Dimension” tends to emphasize a geometric connotation, while “feature” does not.

4 In the model, nonperceptually separable dimensions are effectively treated as a single information source, as it is not possible to sample one without the other.

5 However, as SEA considers only the expected utility of each stimulus feature with regards to the final choice, it is not influenced by factors such as perceptual salience, which are known to influence human eye movements (Rehder & Hoffman, 2005b). Perceptual salience could be included as an independent factor that increases the likelihood of sampling or could instead be reflected in the cost of sampling a feature.

6
Raiffa (1961) describes *terminal* analysis as the selection of an appropriate action after an experiment has been performed, and *preposterior* analysis as the selection of appropriate experiments.

7 A positive blood test for a specific pathogen, for example, can be more informative than a null result.

8 This particular example corresponds to the *probability gain* sampling norm, which provides a compelling account of human information search (Nelson, 2005; Nelson et al., 2010).

9 As this partially observable Markov decision process (POMDP) can only be solved for relatively simple problems (Knox et al., 2012), nonheuristic approaches are often computationally intractable.

10 Development of inaccurate representations can also be driven by similarity between information sources. For instance, information sources that resemble others that impose high costs might be avoided. They can also result from learned selective attention (e.g., “blocking”; Kruschke & Blair, 2000).

11 This process is similar to that of infinite mixture models (Rasmussen, 2000) and Dirichlet-process mixture models (Neal, 2000).

12 Tractable approximations based on particle filters have been developed (e.g., Brown & Steyvers, 2009; Daw & Courville, 2008; Sanborn et al., 2006). Markov Chain Monte Carlo methods (MCMC; Andrieu et al., 2003) can also be used to sample the space of category partitions.

13 The utility of the final action, *a* given each possible state is provided to the model in the form of a utility table (e.g., Table 1).

14 We assume costs associated with the final action are included in the utility table (e.g., Table 1). These costs are not learned by the model, but are specified to match characteristics of the stimulus. Although we do not manipulate costs here, different stimulus features could have different associated costs.

15 In this example, a depth-two search process would involve considering the expected utility of 18 stimulus configurations: [0, ?, ?], [1, ?, ?], [?, 0, ?], [?, 1, ?], [?, ?, 0], [?, ?, 1], [0, 0, ?], [0, 1, ?], [1, 0, ?], [1, 1, ?], [0, ?, 0], [0, ?, 1], [1, ?, 0], [1, ?, 1], [?, 0, 0], [?, 0, 1], [?, 1, 0], [?, 1, 1].

16 The feature-wide parameters, α_0_, determine the likelihood that distinct clusters with the same feature value will be created. Low values of the α_0_ parameter for the category label, for instance, would mean that distinct clusters with the same label would be unlikely.

17 The structure of the Type III problem mirrors that of the Blair et al. (2009) experiment. Although each dimension is potentially relevant, after observing the first (“indicator”) dimension, only one of the two remaining dimensions is relevant. Accordingly, only two dimensions need to be sampled on any given trial.

18 The Type III, IV, and V problems are typically equivalent in difficulty.

19 Model predictions for all six problems are illustrated in Appendix B (Figure B1).

20 Like all other parameters, information costs were held constant across simulations.

21 Interestingly, this category structure is closely related to the Type III category structure of Shepard et al. (1961).

22 Because the calculations of the concept-learning component are stochastic, and because the stimuli were randomized, the yoked model was unable to perform stimulus-specific sampling. When coupled, the probabilistic calculations of the concept-learning component guide active sampling.

23 Like Rehder and Hoffman (2005a), Nelson and Cottrell (2007) considered only these four problems.

## A

**Table:**

Variable	Definition
*a*	Action
α_*j*_	Dirichlet prior for particular value on particular stimulus dimension
α_0_	Dirichlet prior for a particular stimulus dimension
*c*	Coupling probability
*C*_*ij*_	Number of times value *j* has been observed on dimension *i* in a particular cluster
*G*	Expected gain in utility
*F*	Features (or “Dimensions”)
*j*	Feature value
*O*	Indices of observed (i.e., previously sampled) dimensions
*k*	Cluster number
*ℒ*	Sampling cost associated with particular dimensions
*n*_*k*_	Number of items assigned to cluster *k*
*i*	Index of dimension considered for sampling
*U*	Utility

## B

[Fig fig9]
